# Epidermal turnover and iron metabolism in senile lentigo

**DOI:** 10.1111/1346-8138.17034

**Published:** 2024-01-13

**Authors:** Mikiko Odawara, Minori Mezaki, Tomohisa Yoshimura, Akiko Takaoka, Fumino Oda, Hidehisa Saeki, Yoko Funasaka

**Affiliations:** ^1^ R&D Laboratories, Self‐Medication, Taisho Pharmaceutical Co. Ltd. Saitama Japan; ^2^ Department of Dermatology Nippon Medical School Tokyo Japan

**Keywords:** epidermal turnover, iron metabolism, melanin deposition, senile lentigo, skin

## Abstract

Senile lentigo (SL) is a pigmentary disorder associated with disrupted epidermal turnover. Trace minerals in the skin are known to regulate keratinocyte proliferation and differentiation. To clarify the role of iron in SL, we compared the expression of molecules related to iron metabolism between SL lesion (lesion) and the surrounding normal skin (nonlesion). Our results revealed that proteins involved in iron uptake and utilization such as transferrin receptor 1, iron regulatory protein 1, mitoferrin 1, and divalent metal transporter 1 were expressed in the lower epidermis in the nonlesion, while expression of them was also observed in the upper epidermis in the lesion. Ferroportin (FPN), involved in iron export, was expressed in the upper epidermis in the nonlesion, but was only scarcely expressed in the upper epidermis in the lesion. Hepcidin, which promotes FPN degradation, was expressed in the lower epidermis in the nonlesion; however, its expression was also observed in the upper epidermis in the lesion. These changes in the expression of molecules involved in iron uptake/export/utilization might reflect the altered iron utilization state in SL, resulting in disruption of keratinocyte differentiation and disturbing epidermal turnover. Our results suggest that the metabolism of iron in keratinocytes in SL differs from that in the normal epidermis, and these changes could be associated with the abnormal epidermal turnover and decreased melanin excretion in SL.

## 1 INTRODUCTION

Senile lentigo (SL) is characterized by hyperpigmentation, but in some cases it is accompanied by epidermal thickening.^1^ Proliferation and differentiation of keratinocytes in the epidermal layer are strictly regulated to maintain continuous skin renewal (referred to as “turnover”). The basal proliferative keratinocytes migrate to the body surface and differentiate into corneocytes to complete the final differentiation and are then shed. Melanin synthesized in the melanocytes is incorporated into the keratinocytes and then degraded or exfoliated as the stratum corneum.^2^, ^3^ Thus, keratinocyte turnover is an important factor for melanin removal, and the disruption of epidermal proliferation and differentiation can be considered as the cause of epidermal hyperplasia and melanin deposition in SL. Therefore, we investigated the mechanism of epidermal thickening and pigmentation by examining the abnormal turnover caused by dysregulation of iron metabolism. Iron is required for adenosine triphosphate generation in the mitochondria for cell proliferation and has been shown to be involved in the process of keratinocyte proliferation and differentiation in the epidermis.^4^, ^5^ Previous studies have shown that the epidermal iron content is highest in the basal layer, which is the site of proliferation of keratinocytes.^5^ Iron metabolism in the skin is very finely tuned and numerous proteins involved in this process have been identified.^6^ Recently, Asano et al. demonstrated the iron recovery system in the human epidermis.^7^ They demonstrated that molecules participating in iron import and storage were expressed in the lower epidermis, while those mediating iron export were expressed in the upper epidermis. However, no relationship has been reported between changes in the expression of these molecules and the pathophysiology of SL. Therefore, we hypothesized that abnormal localization and metabolism of iron may be involved in the pathogenesis of hyperplasia and melanin deposition in SL, and comparatively analyzed the expression of the molecules involved in iron transport and utilization between tissues of SL lesions (hearafter referred to as “lesions”) and those of the surrounding normal skin.

## 2 METHODS

See Appendix S1.

## 3 RESULTS

**FIGURE 1:**
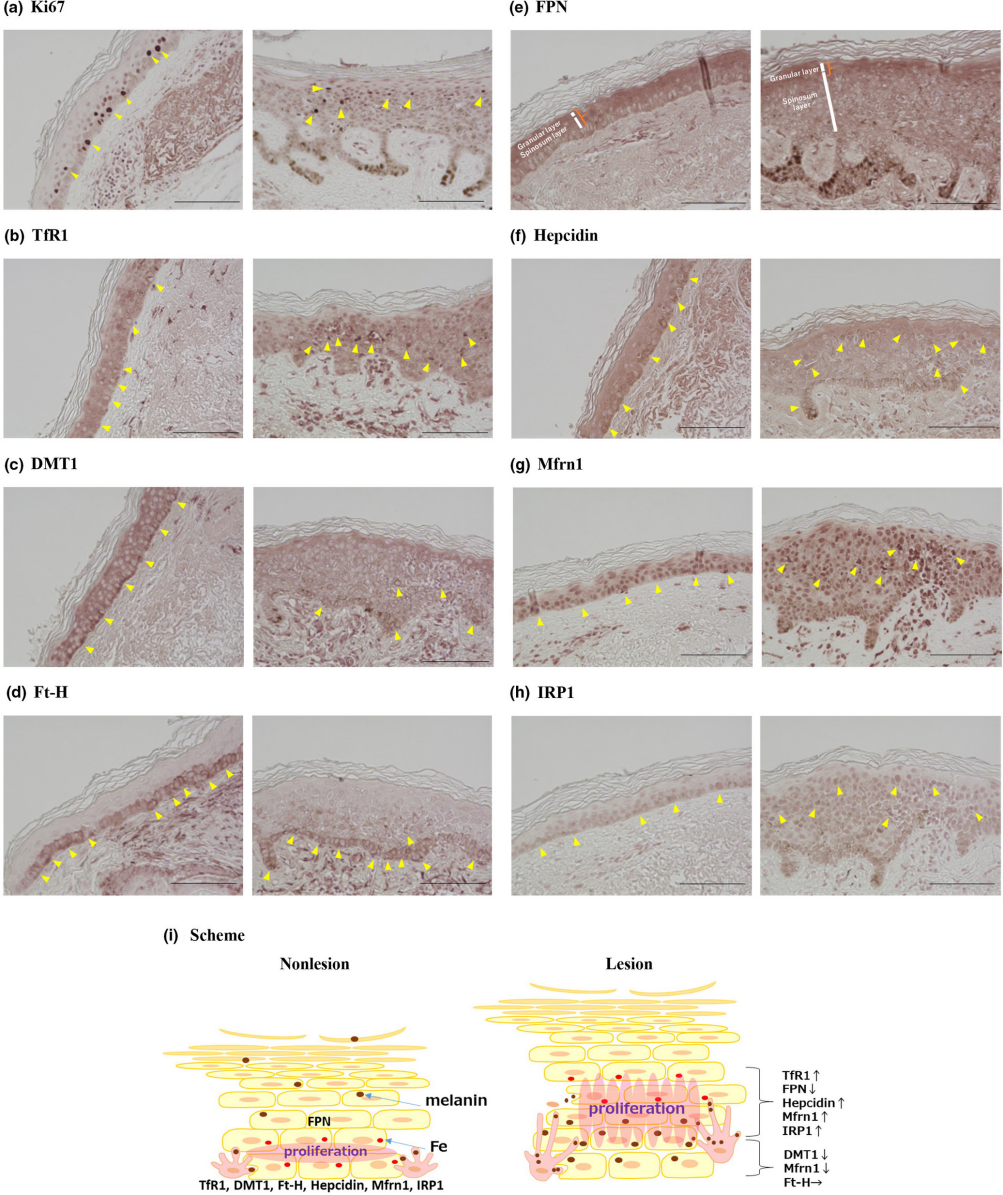
The altered expression of molecules involved in cell proliferation and iron metabolism in the senile lentigo (SL) lesions. (Scale bars, 100 μm). Left, in the nonlesions. Right, in the SL lesions. Immunohistochemistry results of Ki67 (a), transferrin receptor 1 (TfR1) (b), divalent metal transporter 1 (DMT1) (c), ferritin heavy chain (Ft‐H) (d), ferroportin (FPN) (e), hepcidin (f), mitoferrin 1 (Mfrn1) (g), and iron regulatory protein 1 (IRP1) (h). Scheme (i). Arrowheads show positively stained cells. Curly braces show positively stained legions (FPN). In the nonlesions, proliferating suprabasal keratinocytes were observed with iron load. In the SL lesions, altered distribution of iron might have delayed the turnover of melanin‐containing keratinocytes.

We compared the expression intensities of molecules between lesions and the surrounding normal skin (hereafter referred to as “nonlesions”) in the same section. The molecules examined in this study are listed in Table 1. In the nonlesions, Ki67 was expressed in the suprabasal layer, whereas in the SL lesions its expression in the suprabasal layer was decreased in seven of 10 specimens (Figure 1a). Unevenly stained scattered cells were observed in the spinosum layer in the area of the papillomatous growth (nine of 10).

**TABLE 1:** Epidermal expression of each molecule in nonlesions.

Molecules	Key function	Staining results for 10 samples			
		Area of expression	Expression intensity (numbers of samples)		
			Strong	Medium	No/unclear
Ki67	Proliferation	Lower epidermis (suprabasal layer)	10	0	0
TfR1	Transferrin import	Lower epidermis	3	7	0
DMT1	Divalent ion transport	Lower epidermis	3	4	3
Ft‐H	Iron store	Lower epidermis (suprabasal layer)	10	0	0
FPN	Iron export	Lower–upper epidermis (basal, spinosum, granular layers)	5	4	1
Hepcidin	FPN degradation Inhibition of iron export	Lower epidermis	4	4	2
Mfrn1	Iron transport into mitochondria	Lower epidermis	0	9	1
IRP1	TCA cycle activation Iron sensor	Lower epidermis	7	3	0

Abbreviations: DMT1, divalent metal transporter 1; FPN, ferroportin; Ft‐H, ferritin heavy chain; IRP1, iron regulatory protein 1; Mfrn1, mitoferrin 1; TCA, tricarboxylic acid; TfR1, transferrin receptor 1.

The expressions of molecules involved in iron trafficking and iron utilization, such as transferrin receptor 1 (TfR1), divalent metal transporter 1 (DMT1), ferroportin (FPN), hepcidin, mitoferrin 1 (Mfrn1), iron regulatory protein 1 (IRP1), and in iron storing ferritin heavy chain (Ft‐H) were examined. In the nonlesions, TfR1, DMT1, Ft‐H, and hepcidin were expressed predominantly in the basal and suprabasal layers (Figure 1b–d,f). FPN was expressed predominantly in the spinosum and granular layers, with some expression on the cell membrane in the basal layer (Figure 1e). In the lesions, DMT1 expression was decreased (eight of 10) in the lower epidermis, and TfR1 expression was increased in the spinosum layer (eight of 10). The expression of Ft‐H was maintained in the basal layer, but, in addition, it was also observed in the spinosum layer. The expression of FPN in the spinosum layer was decreased (eight of 10) and that of hepcidin was increased in the spinosum and granular layers (seven of 10). The expression of Mfrn1 in the basal layers was decreased (five of 10) and was increased in the spinosum and granular layers (six of 10) in the lesions (Figure 1g). The expression of IRP1 was also increased in the spinosum and granular layers (seven of 10) in the lesions (Figure 1h). The results of these molecular changes are summarized in Table 2.

**TABLE 2:** Epidermal expression of each molecule in the SL lesions: comparison of its intensity with that in the nonlesions.

Molecules	Funciton	Area of expression	Intensity compared to the nonlesion. (numbers of samples)			Interpretation
			Decreased	Unchanged	Increased	
Ki67	Proliferation	Upper epidermis (spinosum layer)	0	1	9	Upper epidermis↑
		Lower epidermis (suprabasal layer)	7	3	0	
TfR1	Import	Upper epidermis (spinosum layer)	0	2	8	Upper epidermis↑
		Lower epidermis	3	7	0	
DMT1	Import	Upper epidermis	0	8	2	Lower epidermis↓
		Lower epidermis	8	2	0	
Ft‐H	Store	Upper epidermis	0	8	2	No significant change
		Lower epidermis (suprabasal layer)	2	8	0	
FPN	Export	Upper epidermis (spinosum layer)	7	3	0	Upper epidermis↓ Lower epidermis↓
		Lower epidermis (basal layer)	8	2	0	
Hepcidin	Export	Upper epidermis	0	3	7	Upper epidermis↑
		Lower epidermis (suprabasal layer)	2	6	2	
Mfrn1	Utilization	Upper epidermis	0	4	6	Upper epidermis↑
		Lower epidermis	5	5	0	
IRP1	Utilization	Upper epidermis	0	3	7	Upper epidermis↑
		Lower epidermis	3	7	0	

Abbreviations: DMT1, divalent metal transporter 1; FPN, ferroportin; Ft‐H, ferritin heavy chain; IRP1, iron regulatory protein 1; Mfrn1, mitoferrin 1; SL, senile lentigo; TfR1, transferrin receptor 1.

## 4 DISCUSSION

Based on these results, we propose the following mechanisms underlying the excessive melanin deposition in SL, as shown in the Scheme (Figure 1i). In the normal skin, molecules involved in iron uptake and utilization were abundantly expressed in the lower epidermis (TfR1, DMT1, IRP1, Mfrn1), which was the site of keratinocyte proliferation. FPN was expressed in the upper epidermis and expelled intracellular iron during differentiation. In the SL lesions, the expression of these molecules was altered, and keratinocyte proliferation was disorganized.

In this study, we demonstrate that scattered Ki67‐positive cells were unevenly stained over a broad area, reflecting a papillomatous growth pattern. This result suggests that the normal turnover of the epidermis was disrupted in the thickened areas of SL. Our results of the expression of TfR1, DMT1, and FPN in the nonlesions were consistent with those of Asano et al., who reported higher iron uptake activity in the lower epidermis and higher iron discharge activity in the upper epidermis. This mechanism was thought to lead to higher intracellular iron concentration in the lower epidermis. In addition, to our knowledge, we showed for the first time the expression of Mfrn1 which plays a role in iron transport into mitochondria in the keratinocytes of the lower epidermis, indicating that cellular iron was used in mitochondria.

In contrast, in the lesions, while the expression of the molecules involved in iron uptake and utilization was decreased in the lower epidermis (DMT1, Mfrn1), they were increased in the upper epidermis (TfR1, IRP1, Mfrn1). Furthermore, the expression of the molecule involved in iron export was attenuated in the upper epidermis (FPN), indicating that iron utilization was increased in the upper epidermis in the lesions. An increased expression of hepcidin in association with a decreased expression of FPN was noted in the spinosum layer. Hepcidin is a key regulator of the entry of iron into the circulation in mammals. It binds to the FPN, triggers its degradation, and reduces FPN‐mediated iron efflux. The expression pattern of hepcidin suggests that hepcidin may be involved in the regulation of iron utilization in the epidermis. Hepcidin is produced by the liver in response to infection and chronic inflammation. In the skin, its expression has been reported to be increased in patients with leprosy and other infections, but the association with skin pigmentation has not been examined to date.^6^, ^8^, ^9^ In this study, we found alteration of the hepcidin expression with inverse correlation with FPN expression. This suggests that the decreased expression of FPN could be mediated by the action of hepcidin. Altered expression of these molecules may inhibit keratinocyte differentiation, resulting in reduced epidermal cell turnover in SL.

The current study shows that increased iron utilization can be involved in the abnormal proliferation and differentiation of keratinocytes in SL. Normalizing abnormalities of iron metabolism might improve the perturbed turnover and melanin accumulation in SL.

## CONFLICT OF INTEREST STATEMENT

This study was performed as joint research between Taisho Pharmaceutical Co., Ltd. and Nippon Medical School and founded by Taisho Pharmaceutical CO., Ltd. M.O., M.M., T.Y., and A.T. are employees of Taisho Pharmaceutical CO., Ltd. H.S. is an editorial board member of *The Journal of Dermatology* and a co‐author of this article. To minimize bias, they were excluded from all editorial decision‐making related to the acceptance of this article for publication.

## Supporting information


Appendix S1.
