# Supplementary material for: Epidermal turnover and iron metabolism in senile lentigo
Source: J Dermatol. 2024 Jan 13;51(3):453–7. doi: 10.1111/1346-8138.17034 (PMC11484136; doi:10.1111/1346-8138.17034)
Supplement: Supplementary file 1 — Appendix S1. [file JDE-51--s001.docx]

**Appendix S1**

**METHODS**

**Human skin samples**

We collected 10 specimens containing both SL lesions and surrounding normal skin from the subjects. All the specimens were obtained from sun-exposed areas (head or neck) of of the patients who visited Nippon Medical School Hospital between January 1, 2015 and March 31, 2018. None of the donor patients suffered from anemia of iron deficiency. All individuals in this study provided informed consent complied with the ethical principles in the Declaration of Helsinki. This study was conducted with the approval of the Ethics Committee of Nippon Medical School Hospital.

**Immunohistochemistry**

Formalin-fixed paraffin-embedded tissue samples were cut at 4 μm thickness for IHC (immunohistochemistry). The samples were deparaffinized, followed by incubation with the primary antibodies raised in a rabbit overnight at 4°C. Antigen retrieval was performed for Ki67, TfR1, IRP1 and Mfrn1. Visualization was performed using a peroxidase-conjugated anti-rabbit/mouse immunoglobulin (LSAB2 kit; K0675, DAKO, Carpenteria, CA, USA) and the substrate (Vector NovaRED^®^ substrate kit; SK-4800, Vector Laboratories, Burlingame, CA, USA). Control for the specificity of the immunoperoxidase reactions was established by replacing the primary antibodies with a rabbit IgG fraction (X0903; DAKO, Carpenteria, CA, USA).

As primary antibodies, we used Ki67 (ab15580), divalent metal transporter 1 (DMT1) (ab140977), ferroportin (FPN) (ab78066), ferritin heavy-chain (Ft-H) (ab65080), iron regulatory protein1 (IRP1) (ab62701) and mitoferrin 1 (Mfrn1) (ab102959), all of which were purchased from Abcam (Cambridge, UK). The antibody to transferrin receptor 1 (TfR1) (HPA028598) was purchased from Atlas Antibodies (Stockholm, Sweden).

**Observation and analysis**

The localization and staining intensities were compared between the SL lesions and the surrounding normal skin in each specimen. Expression changes were defined by similar trends of differences noted in a minimum of 5 specimens, with no specimens showing contradictory alterations.
